# A Meta-Analysis of the Effects of Tailor Activity Program (TAP) for People with Dementia

**DOI:** 10.1093/geroni/igab046.3354

**Published:** 2021-12-17

**Authors:** Jiin Jeong, Eun-Young Yoo, Byoung-Ho Kang, Yae-Na Ha

**Affiliations:** 1 Graduate school, Yonsei University, Republic of Korea, Kangwon-do, Republic of Korea; 2 College of Software and Digital Healthcare Convergence, Yonsei University, Republic of Korea, Kangwon-do, Republic of Korea

## Abstract

Ninety eight percent of people with dementia are accompanied by neuropsychiatric symptoms (NPS). NPS is an important predictor of the negative prognosis of dementia. It also increases the burden on caregivers and lowers the quality of life. The tailored activity program (TAP), which is occupation-based intervention, have a positive effect on reducing NPS through meaningful activities. The aim of this study was to provide an integrated effectiveness of the TAP on NPS in people with dementia and caregiver burden through meta-analysis. We searched for studies that indicated the effectiveness of TAP through Embase, ProQuest, Pubmed, and RISS. We included a total of seven TAP studies written in Korean and English. Of these seven study designs, five were randomized control trials (RCTs) and two were one group non-RCTs. The result of meta-analysis shows that the effect size of the NPS was 0.62 (95% confidence interval [CI]=0.40-0.83, p<0.001), the caregiver burden was 0.68 (95% CI=0.29-1.07, p=0.001). Both variables indicated moderate effect. These results indicate that the TAP is an effective intervention for reducing NPS of people with dementia and the burden of caregivers. Therefore, TAP is clinically useful approach, we expect TAP to be actively applied to people with dementia in the community.

